# A new rare nematode *Nothocriconemoides hangzhouensis* n. sp. (Nematoda: Criconematidae) from Hangzhou, China

**DOI:** 10.21307/jofnem-2020-006

**Published:** 2020-03-18

**Authors:** Munawar Maria, Wentao Miao, Ruihang Cai, Pablo Castillo, Jingwu Zheng

**Affiliations:** 1 Laboratory of Plant Nematology, Institute of Biotechnology, College of Agriculture & Biotechnology, Zhejiang University, Hangzhou 310058, Zhejiang, P.R. China; 2 Institute for Sustainable Agriculture (IAS), Spanish National Research Council (CSIC), Campus de Excelencia Internacional Agroalimentario, ceiA3, Avenida Menéndez Pidal s/n, 14004 Córdoba, Spain; 3 Ministry of Agriculture Key Lab of Molecular Biology of Crop Pathogens and Insects, Hangzhou 310058, P.R. China

**Keywords:** DNA sequencing, Elm tree, Morphology, Morphometrics, Nematode, New record, Species, Phylogeny, Scanning electron microscopy.

## Abstract

The Family Criconematidae is commonly referred as ring nematodes that include some members with economic importance as plant parasites. During a recent nematode inventory survey at Zhejiang Province, China, a new species of genus *Nothocriconemoides* was detected in the rhizosphere of elm tree. *Nothocriconemoides hangzhouensis* n. sp. can be characterized by the female body having annuli with fine longitudinal striations and 2 to 3 anastomoses at the posterior half of the body. The first cephalic annulus is rounded and expanded enclosing the lip region, and the second annulus is narrow, offset, collar like. *En face* view shows a central elevated labial disk bearing four distinct equal-sized submedian lobes and “I” shaped oral aperture. Excretory pore is located 3–4 annuli posterior to esophageal bulb. Vagina is straight and vulva closed. The ventral side of postvulval annuli is inverted, in majority of individuals. Anus is indistinct and located on the next annuli posterior to vulva. Tail is short, conoid, with forked or branched terminus. Juveniles are devoid of collar-shaped annuli in the lip region. The cephalic region has two rounded annuli where the first annulus shows slight depression in the middle. Body annuli are finely crenated. Anus is indistinct and located 3 to 4 annuli from tail terminus. Tail is short ending in a single lobed terminus. Phylogenetic studies based on analysis of the D2–D3 expansion segments of the 28 S rRNA, ITS rRNA, partial 18 S rRNA, and *cox*I gene revealed that the new species formed a separate clade from other criconematid species, thereby supporting its status as a new species of the genus. The new species showed close relationships with *Discocriconemella sinensis*. Additionally, this is the first record of genus *Nothocriconemoides* from China.

The Criconematidae family is commonly referred as ring nematodes. The family contains 5 subfamilies and 17 genera (Geraert, 2010). Unlike other plant-parasitic nematodes, this group of nematodes has received less attention from nematologists. There are many criconematid genera and species that following formal descriptions are seldom mentioned again in the scientific literature. One such example is the genus *Nothocriconemoides* (Maas et al., 1971). The genus name was derived from the Greek words *nothos* meaning false, *krikos* meaning ring, *nema* meaning nematodes, and *oides* meaning shape (Siddiqi, 2000). The important diagnostic characteristics of this genus include body annuli with fine longitudinal striae making margins that look finely crenated; the second cephalic annulus of female is offset and collar like. Lips have four distinct submedian lobes. Vulva is closed, and anterior lip overhanging in type species. Tail is conoid tapering to acute or sub-acute terminus. Juveniles have crenate annuli and the first annulus is not offset collar like. So far, the genus contains only two species i.e. *Nothocriconemoides crenulatus* (Ivanova, 1984) and *Nothocriconemoides lineolatus* (Maas et al., 1971) that were described from Tadzhikistan and Suriname, respectively. Both species were found associated with forest soils; however, no association has been reported from soils of cultivated areas (Geraert, 2010).

During our nematode inventory survey, a population of *Nothocriconemoides* was detected in the rhizosphere of elm tree. As *Nothocriconemoides* was never reported from China, the present work was undertaken to identify the species status. The morpho-molecular characterization and SEM data of this population were compared with the existing species of the genus. Careful examination revealed that the species under investigation presents unique characteristics and is a new member of the genus *Nothocriconemoides*. Therefore, the paper describes a new *Nothocriconemoides* species with the following objectives: to provide a morphological and molecular characterization of the new species; to elucidate important morphological details through SEM observations; and to study the phylogenetic relationships of these species with other related criconematids species.

## Materials and methods

### Nematode samplings, extraction and morphological study

Nematodes were extracted from soil and root samples using the modified Cobb sieving and flotation-centrifugation method (Jenkins, 1964). For morphometric studies, nematodes were killed and fixed in hot formalin (4% with 1% glycerol) and processed in glycerin (Seinhorst, 1959). The measurements and light micrographs of nematodes were made with a Nikon Eclipse Ni-U 931845 compound microscope. For the SEM examination, the nematodes were fixed in a mixture of 2.5% paraformaldehyde and 2.5% glutaraldehyde, washed three times in 0.1 M cacodylate buffer, post-fixation in 1% osmium tetroxide, dehydrated in a series of ethanol solutions and critical point-dried with CO_2_. After mounting on stubs, the samples were coated with gold with 6 to 10-nanometer thickness and the micrographs were made with 3 to 5 kV operating system (Maria et al., 2018a).

### Molecular analyses

DNA was extracted by transferring individual nematodes into the Eppendorf tube containing 16 μL ddH_2_O. Nematodes were crushed using a sterilized pipette tip, the tubes were centrifuged at 12,000 rpm for 1 min and frozen at −68°C for at least 30 min. Tubes were heated to 85°C for 2 min, and then, 2 μL proteinase K and PCR buffer solution were added. The tubes were incubated at 56°C for 1 to 2 hr and, then, at 95°C for 10 min. After incubation, these tubes were cooled to 4°C and used for conducting PCR (Zheng et al., 2003). Several sets of primers (synthesized by Invitrogen, Shanghai, China) were used in the PCR analyses to amplify the partial 18 S, ITS region, D2–D3 of 28 S of rDNA and partial *cox*I fragments. Primers for amplification of partial 18 S were 18s900–18s1713 (Olson et al., 2017). Primers for amplification of ITS were TW81-AB28 (Joyce et al., 1994). The primers for amplification of D2–D3 of 28 S were D2A and D3B (De Ley et al., 1999). And, finally, the primers used for *cox*I amplification were COI-F5 (5’-AATWTWGGTGTTGGAACTTCTTGAAC-3’) and COI-R9 (5’-CTTAAAACATAATGRAAATGWGCWAC WACATAATAAGTATC-3’) (Powers et al. 2014). PCR conditions were as described by Ye et al. (2007) and Powers et al. (2010). PCR products were evaluated on 1% agarose gels stained with ethidium bromide. PCR products of sufficiently high quality were sent for sequencing by Invitrogen (Shanghai, China).

### Phylogenetic analysis

Newly obtained sequences of *Nothocriconemoides hangzhouensis* n. sp. (D2-D3 expansion segments of 28 S, ITS, partial 18 S rRNA, and partial *cox*I) and the available sequences of other criconematid nematodes obtained from NCBI were used for phylogenetic analyses. Outgroup taxa for the dataset were chosen according to previous published data (Afshar et al., 2019; Maria et al. 2019). Multiple alignments of the different sequences were made using the FFT-NS-2 algorithm of MAFFT v. 7.205 (Katoh and Standley 2013). Sequence alignments were manually visualized using BioEdit (Hall 1999) and edited by Gblocks ver. 0.91b (Castresana 2000) in the Castresana Laboratory server (http://molevol.cmima.csic.es/castresana/Gblocks_server.html) using options for a less stringent selection (minimum number of sequences for a conserved or a flanking position: 50% of the number of sequences +1; maximum number of contiguous non-conserved positions: 8; minimum length of a block: 5; allowed gap positions: with half). Phylogenetic analyses of the sequence datasets were based on Bayesian inference (BI) using MrBayes 3.1.2 (Ronquist et al., 2012). The best-fit model of DNA evolution was obtained using JModelTest V.2.1.7 (Darriba et al. 2012) with the Akaike Information Criterion (AIC). The best-fit model, the base frequency, the proportion of invariable sites, and the gamma distribution shape parameters and substitution rates in the AIC were then given to MrBayes for the phylogenetic analyses. An unlinked general time-reversible model with invariable sites and a gamma-shaped distribution (GTR fo li + G) was used for the D2-D3 expansion segments of 28 S rRNA, ITS, partial 18 S, and partial *cox*I. These BI analyses were run separately per dataset using four chains for 2 × 10^6^ generations for all of the molecular markers. A combined analysis of the three genes was not undertaken due to some sequences not being available for all species. The Markov chains were sampled at intervals of 100 generations. Two runs were conducted for each analysis. After discarding burn-in samples and evaluating convergence, the remaining samples were retained for further analyses. The topologies were used to generate a 50% majority-rule consensus tree. Bayesian posterior probabilities (BPP) are given on appropriate clades. Trees from all analyses were visualized using FigTree software V.1.42 (http://tree.bio.ed.ac.uk/software/figtree/).

## Results and description

### Systematics

**Figure 1:**
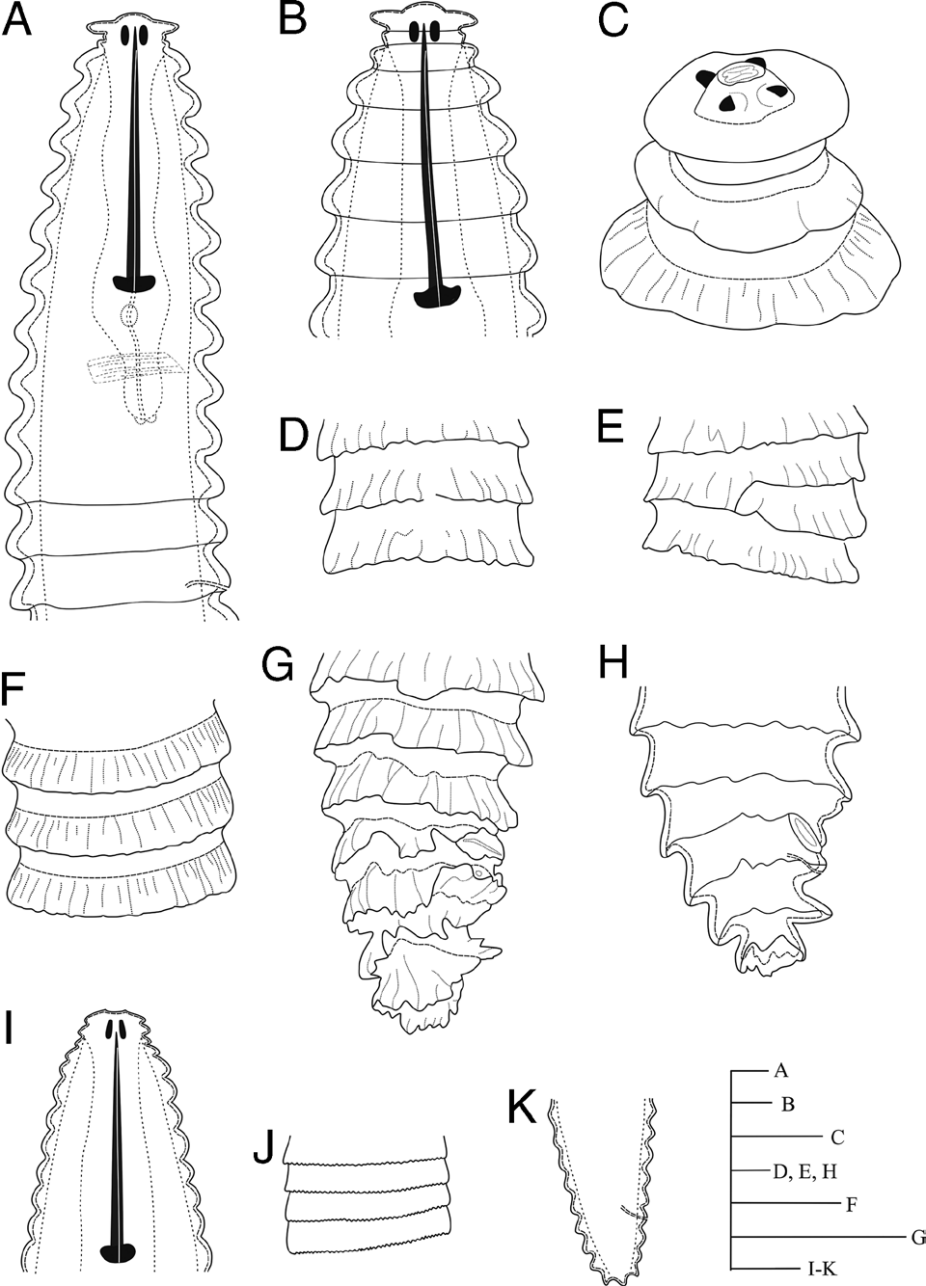
Line drawings of *Nothocriconemoides hangzhouensis* n. sp. Female A: esophageal region; B: Cepahlic region; C: *En face* view: D-F: Cuticle markings; G: Tail region under SEM; H: Tail region under LM; I: Cepahlic region of juvenile: J: Crenation on cuticle of juvenile; K: Tail region of juvenile. (Scale bars = A =50 μm, B-I = 10 μm).

**Figure 2:**
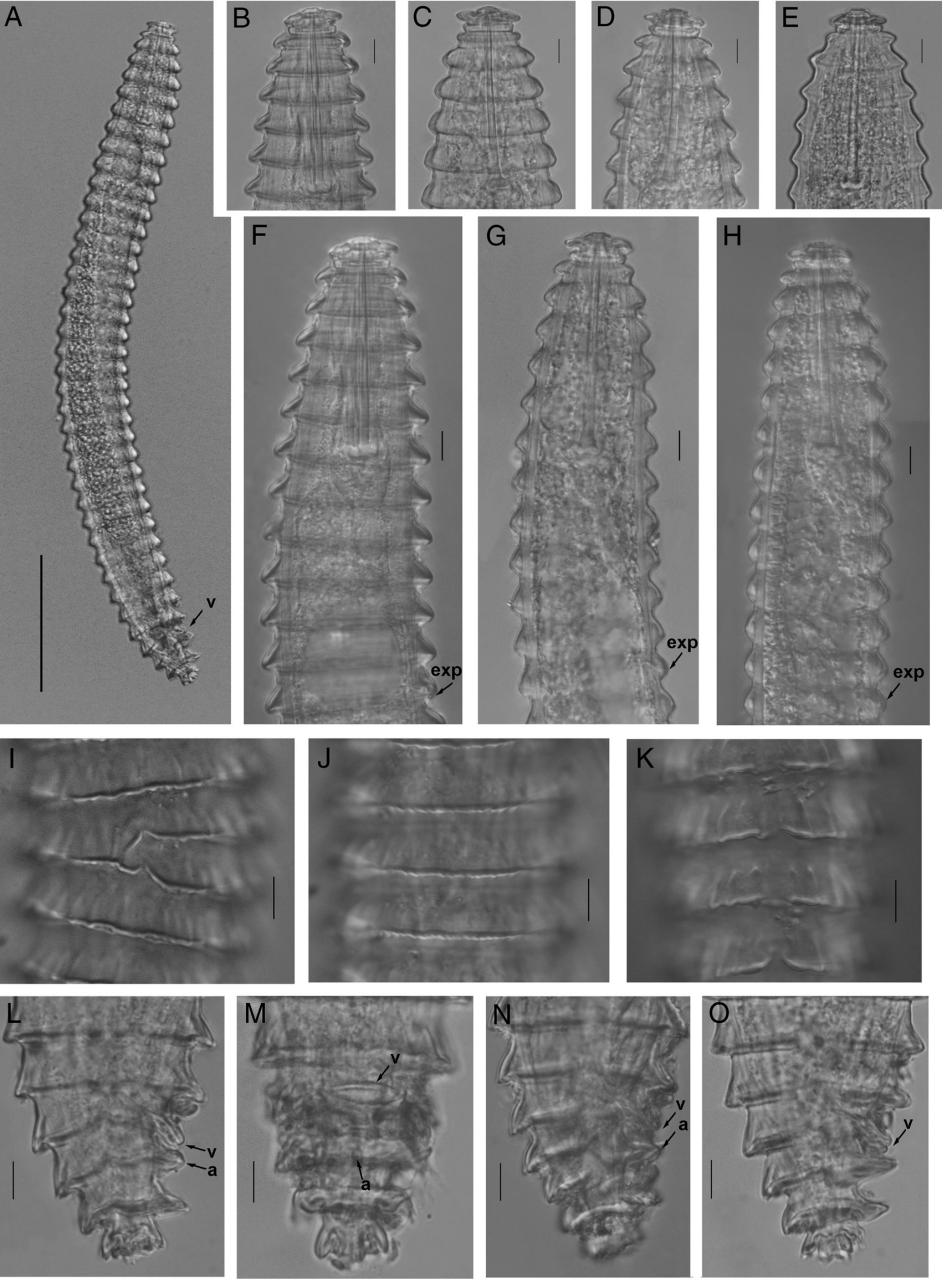
Light photomicrographs of *Nothocriconemoides hangzhouensis* n. sp. Female A: Entire body; B-E: Cepahlic regions; F-H: Esophageal regions, arrow pointing on the excretory pore (exp): I-K: Cuticle markings; L-O: Tail regions, arrows pointing on vulva (v) and anus (a). (Scale bars=A=50 μm, B-I=10 μm).

**Figure 3:**
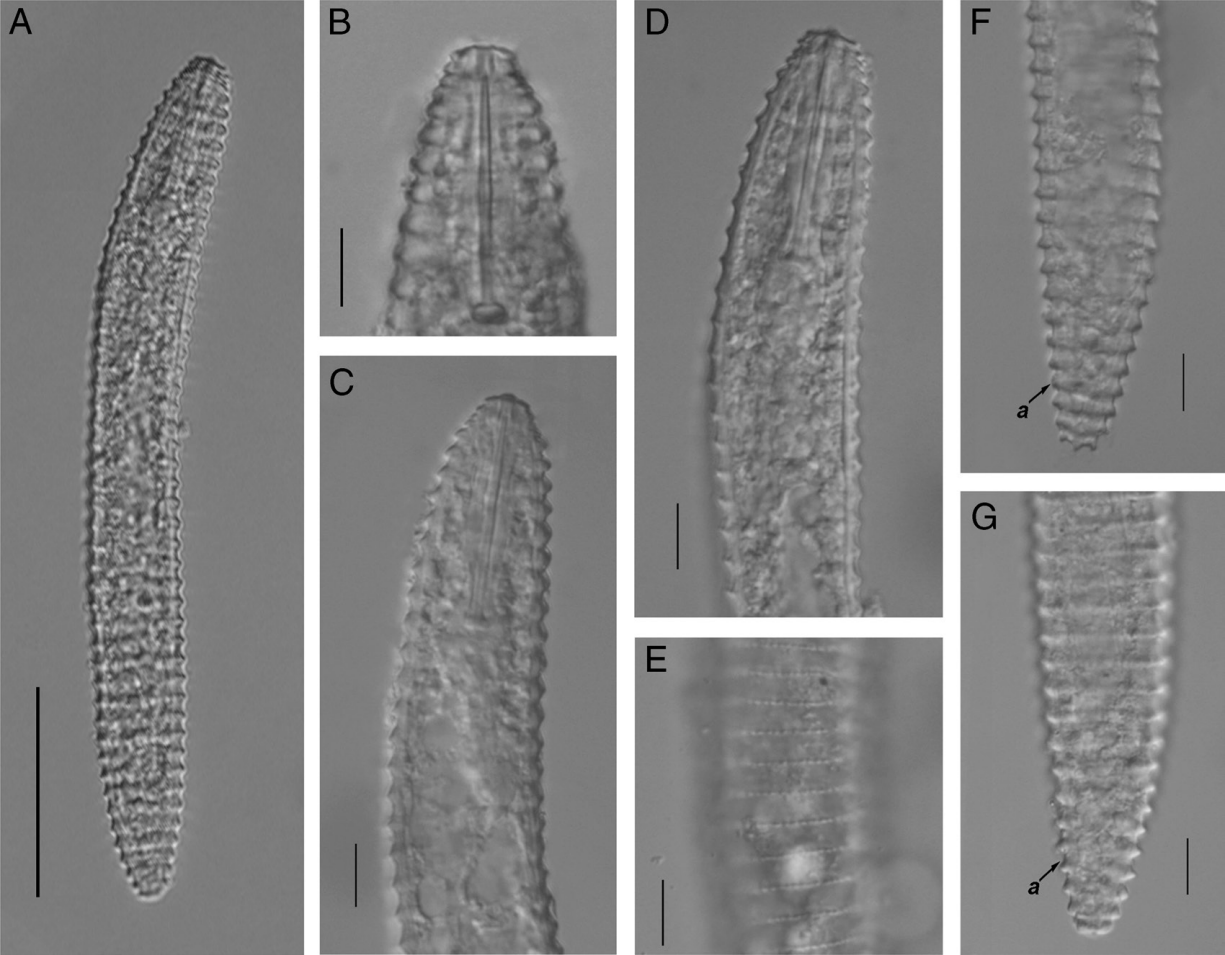
Light photomicrographs of *Nothocriconemoides hangzhouensis* n. sp. Juvenile A: Entire body; B: Cepahlic region; C,D: Esophageal regions: E: Crenation on cuticle; F, G: Tail regions, arrows pointing on anus (a). (Scale bars=A=50 μm, B-I=10 μm).

**Figure 4:**
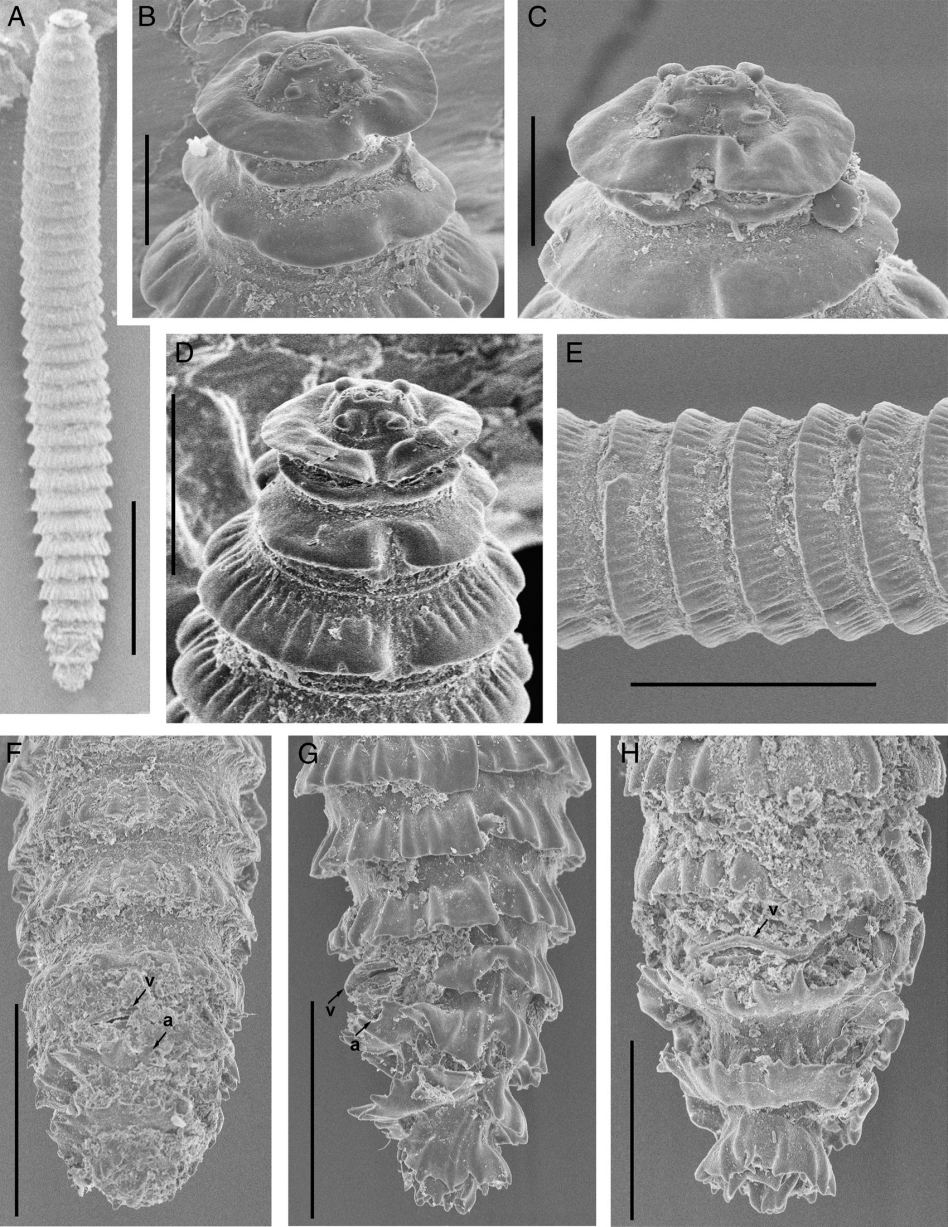
Scanning electron microscopy of *Nothocriconemoides hangzhouensis* n. sp. Female. A: Entire body; B-D: En face view; E: Cuticle markings; F-H: tail regions arrows pointing on vulva (v) and anus (a) (Scale bars, A = 100 μm; B, C = 10 μm; D, H = 20 μm; G-F = 30 μm).

*Nothocriconemoides hangzhouensis n. sp.*

(Figs 1–4; Table 1).

**Table 1.** Morphometric data for *Nothocriconemoides hangzhouensis* n. sp.

	Holotype	Paratype
n		17
Body Length	494.0	487.1±43.8 (419.6-572.3)
R	36.0	37.2±1.2 (35.0-39.0)
Rst	7.0	6.5±0.5 (6.0-7.0)
Rex	15.0	14.7±0.6 (13.0-15.0)
RV	3.0	2.9±0.2 (2.0-3.0)
Rvan	0.0	0.0±0.0 (0.0-0.0)
Ran	3.0	2.9±0.2 (2.0-3.0)
a	8.2	7.9±0.7 (6.3-9.4)
b	4.2	4.1±0.3 (3.5-4.5)
c	18.6	17.3±1.7 (14.2-20.0)
c'	0.8	0.9±0.1 (0.7-1.1)
V	93.2	92.6±0.9 (90.5-94.0)
VL/VB	0.9	1.0±0.1 (0.9-1.2)
Lip height	8.8	9.5±0.7 (7.8-10.6)
Lip diam.	18.4	20.2±1.4 (17.2-22.1)
Stylet length	71.0	71.1±3.0 (64.4-75.5)
Stylet percentage	14.4	14.7±1.2 (13.1-17.4)
Pharynx length	117.5	118.9±5.0 (111.8-129.6)
Body width	60.3	61.8±5.3 (52.0-69. 4)
Vulval body diam.	37.0	35.7±2.5 (31.8-38.5)
Anal body diam.	31.9	32.1±3.1 (26.4-37.4)
Vulva to tail terminus	33.4	36.0±4.0 (29.8-41.7)
Tail length	26.5	28.3±1.9 (23.3-30.5)
Annuli width	13.1	14.5±1.2 (13.1-16.9)

Notes: All measurements are in μm and in the form of mean ± SD (range).

### Description

#### Females

Body is slightly curved ventrally after heat-killing. Body annuli are wide (13-17 μm thick in the middle of the body) with fine longitudinal striations that look like annulus bearing rough cuticular margins. Anastomoses are 2 to 3, located at the posterior half of the body. The first cephalic annulus is rounded and expanded enclosing the lip region. The second annulus narrow, offset, collar like. *En face* view shows a central elevated labial disk bearing four equal-sized submedian lobes and “I”-shaped oral aperture. Stylet is robust with anchor-shaped basal knobs, and DGO indistinct. Esophageal lumen is looped in median esophageal bulb having a medium-sized valvular apparatus. Isthmus is narrow, short, encircled by nerve ring, and basal esophageal bulb distinct. Excretory pore is 3 to 4 annuli posterior to esophageal bulb. Monodelphic gonad is outstretched, and spermatheca is spherical, filled with sperm. Vagina is straight, vulva is closed, and vulval lips do not project above body contour. The ventral side of postvulval annuli is inverted, in majority of individuals. Anus is indistinct and located on the next annuli posterior to vulva. Tail is short and conoid, longitudinal striations are more prominent on the terminal annulus that gives the appearance of forked or branched terminus.

#### Male

Not found.

#### Juveniles (n=5)

Except for the cephalic region, they are similar to females; cephalic region of juveniles are devoid of collar-shaped annuli. Two rounded annuli are present and the first annulus show slight depression in the middle. Body annuli are narrower (4.5-5.5), slightly higher in number R = (48-51) and finely crenated. Stylet is (32.5-36.7) μm long and esophageal components are similar as those of females but less developed. Anus is indistinct and located 3 to 4 annuli from tail terminus. Tail is short and ends in a single lobed terminus.

### Type host and locality

This population was found in the rhizosphere of *Ulmus* sp. from Zijingang Campus, Zhejiang University, Hangzhou, Zhejiang Province, P.R. China, on February 2019. The geographical position of the sampling site is E: 120 °4´54˝ N: 30 °17´5.

### Type material

Holotye female and 13 female paratypes (slide numbers ZJU-30-01-ZJU-30-03) were deposited in the nematode collection of Zhejiang University, Hangzhou, China. Four females and two juveniles paratypes on two slides (Slide numbers T-7353, T-7356) and ten additional females on two slides were (T-7354-55) deposited at USDA nematode collection, Beltsville, Maryland, USA. The Zoobank code is as follows: LSID urn:lsid:zoobank.org:pub:E977B880-9EF3-4E2C-BB29-4BD970D63F0A

### Etymology

The species epithet refers to the City name where the species was detected.

### Diagnosis and relationships

The new species can be characterized by wider body annuli that have fine longitudinal striations that look like annulus bearing rough cuticular margins, and 2 to 3 anastomoses at the posterior half of the body. The first cephalic annulus is rounded and expanded enclosing the lip region, and the second annulus is narrow, offset, collar like. Four prominent submedian lobes are present. Excretory pore is 3 to 4 annuli posterior to esophageal bulb. Vagina is straight and vulva is closed. The ventral side of postvulval annuli is inverted, in majority of individuals. Anus is indistinct and located on the next annuli posterior to vulva. Tail is short, with forked or branched terminus.

The genus only contains two species; It can be differentiated from *N. crenulatus* by having shorter stylet 64.4 to 75.5 vs 87 to 96 μm long, less number of body annuli R = 35.0 to 39.0 vs 60 to 68, less number of annuli between vulva and tail terminus RV = 2.0 to 3.0 vs 6 to 8, location of anus (next annuli to vagina vs 3 to 4 annuli posterior to vagina), and tail terminus morphology (terminal annulus forked or branched vs button shaped).

The new species differs from *N. lineolatus* by less number of body annuli R = 35.0 to 39.0 vs 57 to 64, less number of annuli between vulva and tail terminus RV = 2.0 to 3.0 vs 7 to 9, location of anus (next annuli to vagina vs 3 to 5 annuli posterior to vagina), anastomosis (2-3 vs 1), lip annuli (2 vs 4), submedian lobes (separate as four vs connected as 2 subdorsal and sublateral lobes), position of excretory pore (3 to 4 annuli posterior to esophageal bulb vs at the same level of esophageal bulb), vulval lip ornamentation (absent vs present), and tail terminus morphology (terminal annulus forked or branched vs bifid or irregular).

### Molecular profiles and phylogenetic status

The new species was molecularly characterized using partial 18 S, D2-D3 of 28 S, ITS and *cox*I sequences and obtained sequences were deposited in the GenBank. As this is the sole species of genus *Nothocriconemoide*s with molecular characterization, in order to predict the closely related species all the named criconematid species were included in the phylogenetic analysis.

**Figure 5:**
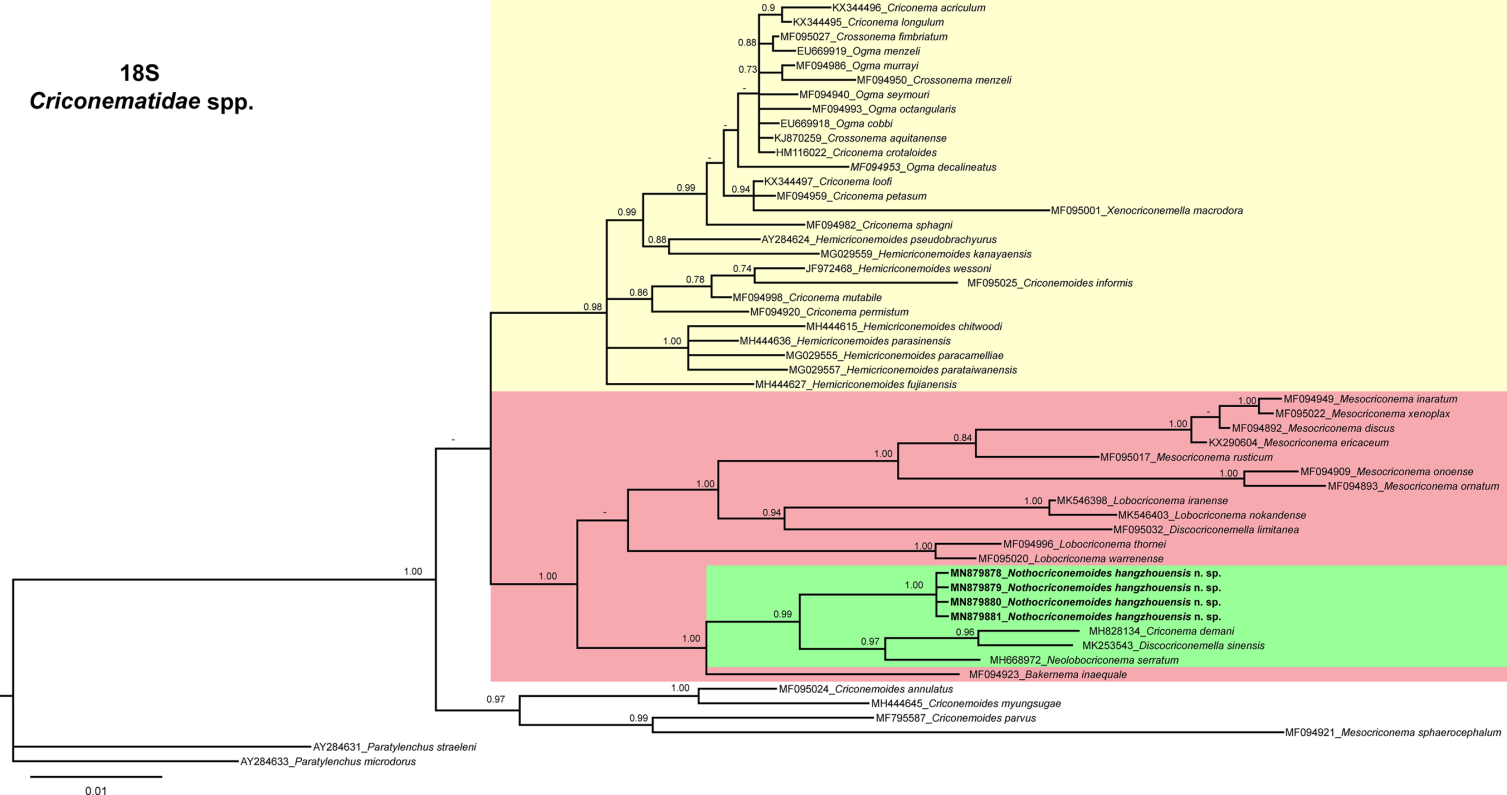
Phylogenetic relationships of *Nothocriconemoides hangzhouensis* n. sp. with other criconematids species as inferred from Bayesian analysis using the 18 S rRNA gene sequence dataset with the GTR + I + G model (−lnL=7,315.8130; AIC = 14,859.6260; freqA=0.2371; freqC=0.2413; freqG=0.2833; freqT=0.2384; R(a)=1.5166; R(b)=2.2509; R(c)=09364; R(d)=0.7246; R(e)=6.0997; R(f)=1.0000; Pinva=0.6630; and Shape=0.6070). Posterior probability more than 70% is given for appropriate clades. Newly obtained sequences are indicated in bold.

In 18 S tree (Fig. 5), *Nothocriconemoides hangzhouensis* n. sp. (MN879878-MN879881) is a sister species of *Criconema demani* (Micoletzky, 1925) (MH828134), *Discocriconemella sinensis* (Maria et al., 2019) (MK253543) and *Neolobocriconema serratum* (Khan and Siddiqi, 1963; Mehta and Raski, 1971) (MH668972). The pairwise sequence identities of the new species with its sister species are 97.53 to 98.9% (5-20 bp difference). This clade is further grouped with *Bakernema inequale* (Taylor, 1936; Mehta and Raski, 1971) (MF094923) and species of *Mesocriconema* (Andrássy, 1965), *Lobocriconema* (De Grisse and Loof, 1965) and *D. limitanea* (Luc, 1959; De Grisse and Loof, 1965) (MF095032).

**Figure 6:**
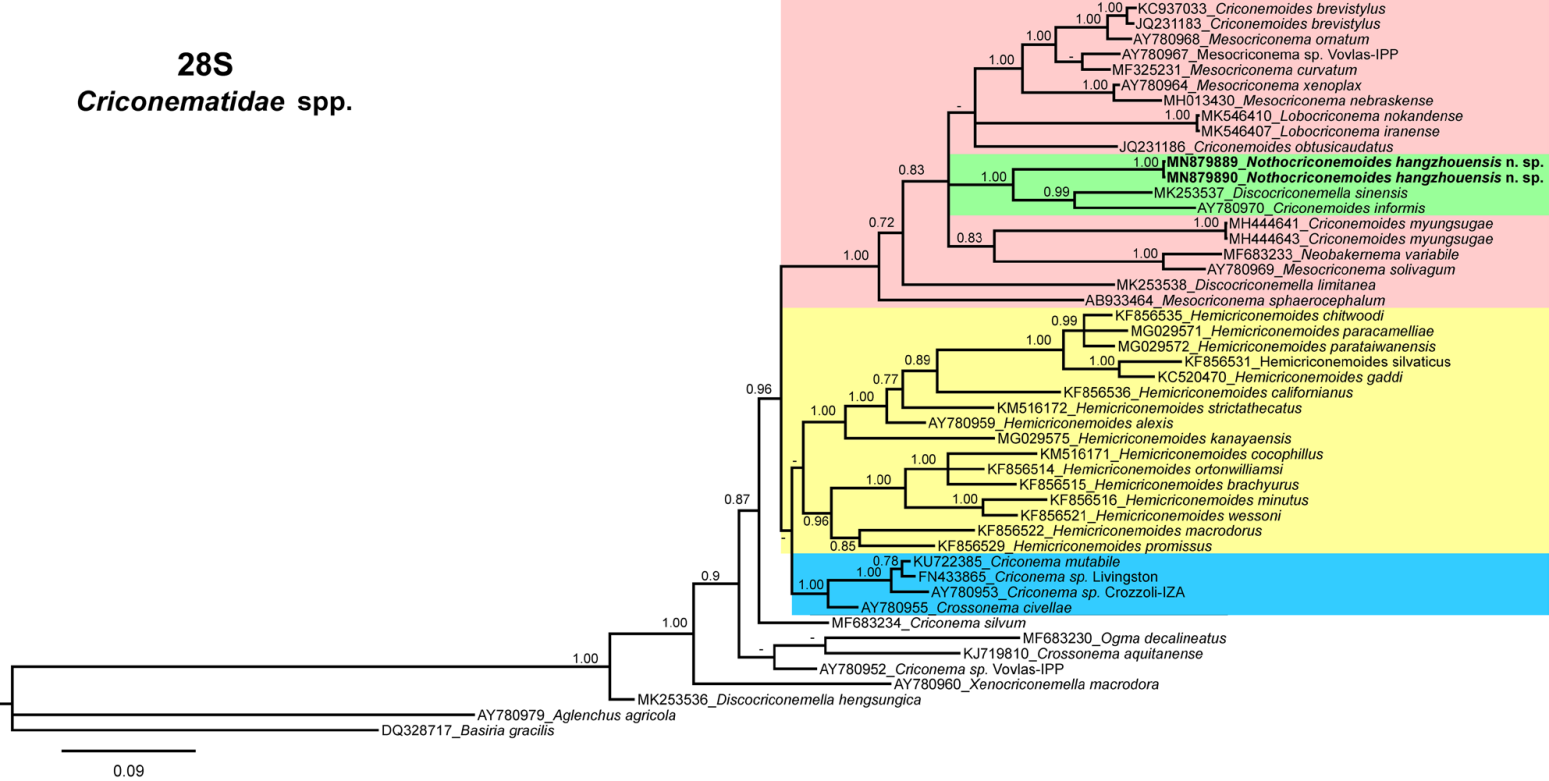
Phylogenetic relationships of *Nothocriconemoides hangzhouensis* n. sp. with other criconematids species as inferred from Bayesian analysis using the D2-D3 of 28 S rRNA gene sequence dataset with the GTR + I + G model (−lnL=8,382.1334; AIC=16,972.2669; freqA=0.1451; freqC=0.2354; freqG=0.3515; freqT=0.2681; R(a)=0.8404; R(b)=2.5613; R(c)=1.6924; R(d)=0.4616; R(e)=4.7092; R(f)=1.0000; Pinva=0.2730; and Shape=0.8370). Posterior probability more than 70% is given for appropriate clades. Newly obtained sequences are indicated in bold.

In 28 S tree (Fig. 6), *Nothocriconemoides hangzhouensis* n. sp. (MN879889-MN879890) clustered with species of *Mesocriconema*, *Criconemoides* (Taylor 1936), *Lobocriconema* and *Neobakernema* (Ebsary 1981) but it is sister species of *Discocriconemella sinensis* (MK253537) and *Criconemoides informis* (Micoletzky, 1922; Taylor, 1936) (AY780970). The pairwise sequence identities of the new species with its sister species are 84.93-89.01% (70-82 bp difference).

**Figure 7:**
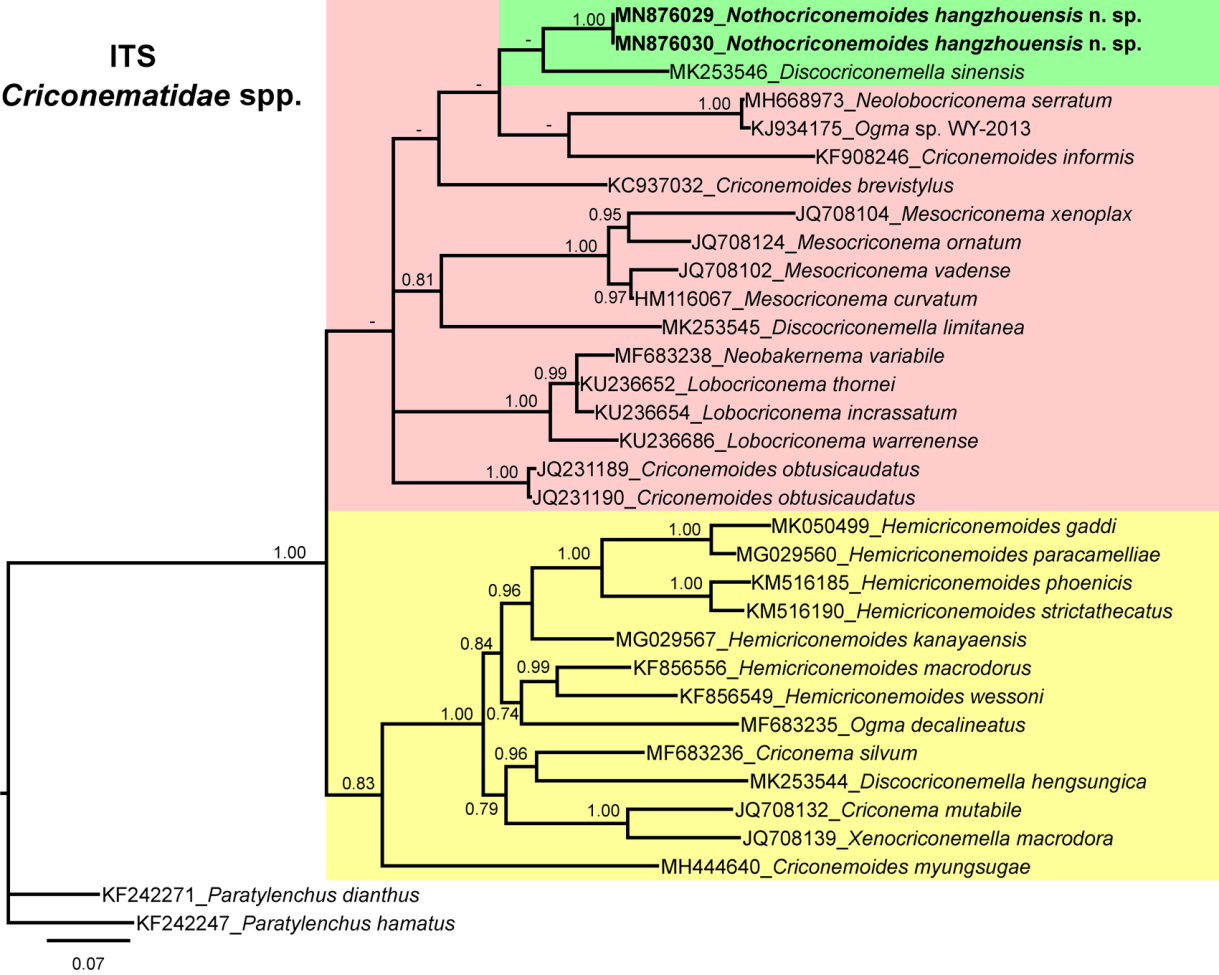
Phylogenetic relationships of *Nothocriconemoides hangzhouensis* n. sp. with other criconematids species as inferred from Bayesian analysis using the ITS rRNA gene sequence dataset with the GTR + I + G model (−lnL=7727.9982; AIC = 15603.9963; freqA=0.2067; freqC=0.2560; freqG=0.2814; freqT=0.2559; R(a)=1.5781; R(b)=2.9918; R(c)=1.7856; R(d)=0.6423; R(e)=2.8799; R(f)=1.0000; Pinva=0.0460; and Shape=0.6180). Posterior probability more than 70% is given for appropriate clades. Newly obtained sequences are indicated in bold.

The majority of criconematid species were not characterized for ITS sequences, and based on the available sequences, the ITS tree (Fig. 7), was constructed. It indicated that *Nothocriconemoides hangzhouensis* n. sp. (MN876029-MN876030) is a sister species of *Discocriconemella sinensis* (MK253546). The sequence similarity between the new species and the sister species is 81.80% (130 bp difference). This clade further grouped with species of *Criconemoides*, *Lobocriconema*, *Mesocriconema*, *Neobakernema* and *D. limitanea*.

**Figure 8:**
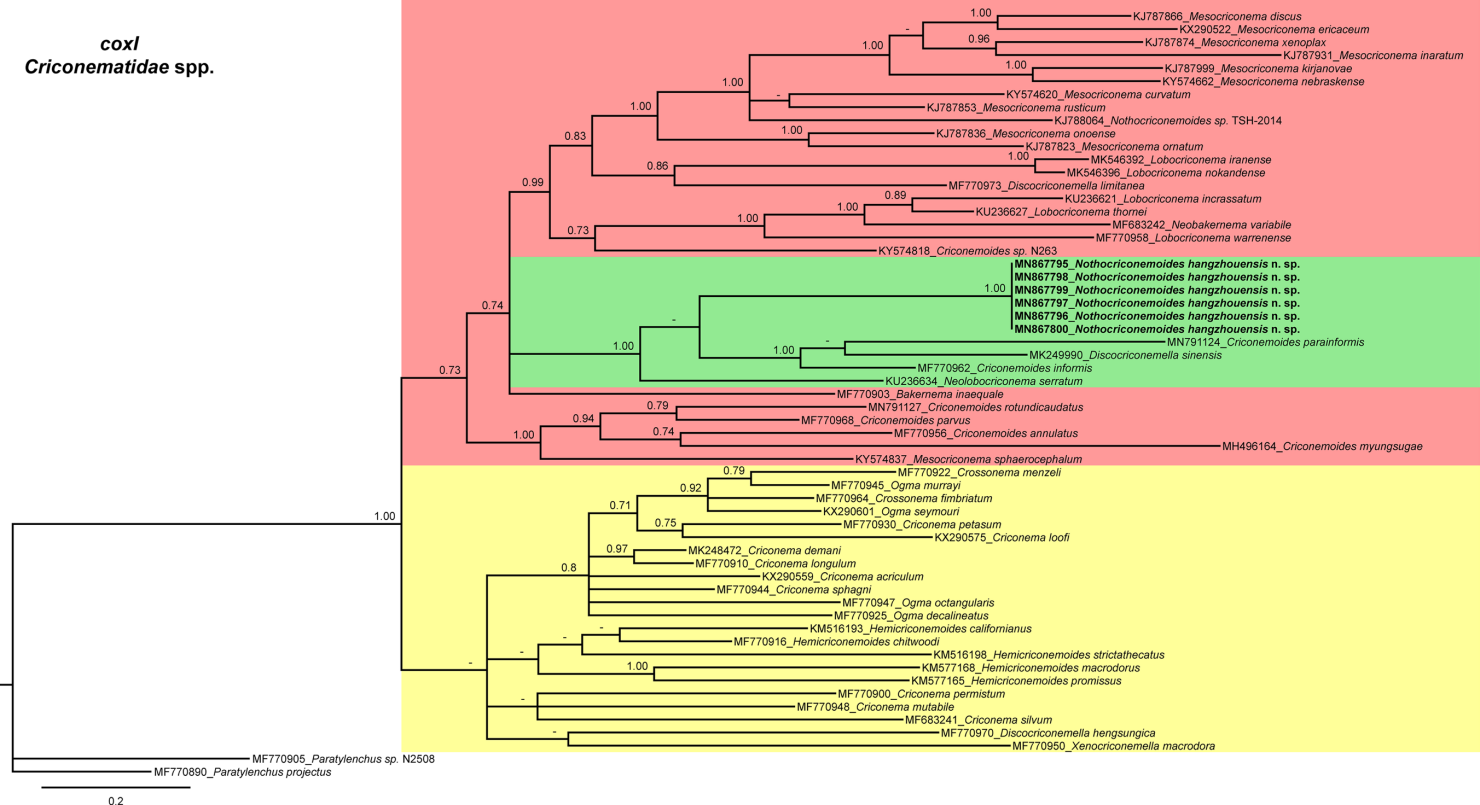
Phylogenetic relationships of *Nothocriconemoides hangzhouensis* n. sp. with other criconematids species as inferred from Bayesian analysis using the *cox*I gene sequence dataset with the GTR + I + G model (−lnL=13,473.0592; AIC=27,198.1184; freqA=0.3715; freqC=0.0509; freqG=0.0477; freqT=0.5299; R(a)=0.7544; R(b)=36.5547; R(c)=1.6680; R(d)=51.5187; R(e)=20.3538; R(f)=1.0000; Pinva=0.2510; and Shape=0.3470). Posterior probability more than 70% is given for appropriate clades. Newly obtained sequences are indicated in bold.

In the *cox*I tree (Fig. 8), *Nothocriconemoides hangzhouensis* n. sp. (MN867795-MN867799, MN867800) clustered with species of the genera *Bakernema*, *Criconemoides*, *Lobocriconema*, *Mesocriconema*, *Neobakernema*, *Neolobocriconema*, but it is a sister species of *Discocriconemella sinensis* (MK249990) and *Criconemoides informis* (MF770692). The pairwise sequence identities of new species with its sister species are 82.7 to 89.0%.

## Discussion

Since 1960, the taxonomy of criconematids has been revised independently and more or less simultaneously by several nematologists, which caused confusions and conflicting definitions of genera as well as contradictions in proposed species synonyms (Subbotin et al., 2005). One such example is *Mesocriconema xenoplax* (Raski, 1952; Loof, 1988), it has been called *Macroposthonia xenoplax* (Siddiqi, 2000; Wouts, 2006), *Criconemella xenoplax* (Xiang et al., 2010), or *Criconemoides xenoplax* (Decraemer and Geraert, 2006; Decraemer and Hunt, 2006; Cid Del Prado Vera and Talavera, 2012).

Criconematids have been widely accepted as a monophyletic group based on the esophageal structure, monodelphic ovary and sexual dimorphism. However, there is no concrete phylogenetic evidence of criconematids subfamily, genus and subgenus grouping (Powers et al., 2017). Geraert (2010) placed *Nothocriconemoides* in the subfamily Macroposthoniinae but phylogenetically the new species grouped with various species of *Bakernema*, *Criconemoides*, *Mesocriconema*, *Neobakernema* (subfamily Macroposthoniinae) *Criconema*, *Lobocriconema*, *Neolobocriconema* (Criconematinae) and *Discocriconemella sinensis* (Discocriconemellinae). *Nothocriconemoides hangzhouensis* n. sp. can be differentiated from *Discocriconemella sinensis* and *Criconema* spp. based on the presence of four distinct submedian lobes and second annulus offset, collar like, from *Bakernema*, *Criconemoides*, *Mesocriconema*, *Neobakernema* based on the presence of fine longitudinal striae on the cuticle, and from *Lobocriconema*, *Neolobocriconema* based on the absence of rows of scales on juveniles.

Additionally, *Nothocriconemoides hangzhouensis* n. sp. appeared as a sister species of *Discocriconemella sinensis* in our phylogenetic analysis. *Discocriconemella* species are characterized by the presence of cephalic disc (Geraert, 2010), and currently the genus contains 29 species but only *D. hengsungica* (Choi and Geraert, 1975), *D. limitanea* and *D. sinensis* are molecularly characterized; interestingly, none of these species display monophyletic behavior (Maria et al., 2018b, 2019). *Nothocriconemoides hangzhouensis* n. sp. has a large labial annulus resembling the *Discocriconemella* type 1 cephalic disc (round to oval with uninterrupted margins) of *sensu* Vovlas (1992). It is also noted that *Discocriconemella* species having type 3 (disc intend medially and laterally giving a four-lobed appearance) cephalic disc is not easy to differentiate from *Mesocriconema* (Geraert, 2010). Several authors have expressed their concerns that *Discocriconemella* species showed considerable variation in distinguishing characters (Orton Williams, 1981; Vovlas, 1992; Siddiqi, 2000). It is likely that a large labial disc is a homoplastic character that independently appears in several criconematids lineages. To this point, we only assume that close phylogenetic relationship between *Nothocriconemoides hangzhouensis* n. sp. and *D. sinensis* is mainly because of a similar arrangement of the labial annulus, except the submedian lobes. We agreed with Jahanshahi-Afshar et al. (2019) that majority of criconematids genera and species have yet to be sequenced, and with the inclusion of additional/new sequences of criconematids, the phylogenetic studies could provide better insights than now.

*Nothocriconemoides hangzhouensis* n. sp. is the first-named species of this genus to be molecularly characterized. In our *cox*I tree, an unknown *Nothocriconemoides* sp. (KJ788064) from Costa Rica is arranged distantly from *Nothocriconemoides hangzhouensis* n. sp. When the information attached to this unidentified species is examined (at https://nematode.unl.edu/sp-16137.htm), it is observed that this population does not fit with the generic definition of *Nothocriconemoides* i.e. the second cephalic annulus of female is offset collar like, lips have four distinct submedian lobes and vulva is closed. Uncertainties concerning the correct identity of some GenBank sequences and lack of sufficient ultra-morphological characterization presenting challenges in the taxonomy of criconematids. The phylogeny of the majority of criconematids taxa is not well resolved, and to this point, we only suggest that molecular identification can be an efficient way of identifying species; however, linking the correct molecular information to the detected species is an important aspect. The generic status of new species is assigned primarily on the basis of morphological characters of females and juveniles. This is the first report of the genus *Nothocriconemoides* from China.

## References

[ref001] Andrássy I. 1965 Verzeichnis und Bestimmungsschlussel der Arten der Nematoden Gattungen *Criconemoides* Taylor, 1936 und *Mesocriconema* n. gen. Opuscula Zoologica, Budapest 5:153–171.

[ref002] Afshar F. J., Pourjam E. and Pedram M. 2019 New morphological observations on *Neolobocriconema serratum* (Khan & Siddiqi, 1963) Mehta & Raski, 1971 (Rhabditida: Criconematidae). Nematology 21:419–434.

[ref003] Choi Y. E. and Geraert E. 1975 Criconematids from Korea with the description of eight new species (Nematoda: Tylenchida). Nematologica 21:35–52.

[ref004] Castresana J. 2000 Selection of conserved blocks from multiple alignments for their use in phylogenetic analysis. Molecular Biology and Evolution 17:540–552. 10742046 10.1093/oxfordjournals.molbev.a026334

[ref005] Cid Del Prado Vera I. and Talavera M. 2012 Criconematoidea In Manzanilla-Lopez R. H. and Marban Mendoza N. (Eds). Practical Plant Nematology. State of Mexico, Mexico: Basic Library of Agriculture, pp. 479–519.

[ref006] Darriba D., Taboada G. L., Doallo R. and Posada D. 2012 jModelTest 2: more models, new heuristics and parallel computing. Nature Methods 9:772. 10.1038/nmeth.2109 PMC4594756 22847109

[ref007] De Grisse A. T. and Loof P. A. A. 1965 Revision of the genus *Criconemoides* (Nematoda). Overdruk uit de Mededelingen van Landbouwhoeschool en de Opzoekingsstations van de Staat Gent 30:577–603.

[ref008] De Ley P., Félix M. A., Frisse L. M., Nadler S. A., Sternberg P. W. and Thomas W. K. 1999), Molecular and morphological characterization of two reproductively isolated species with mirror-image anatomy (Nematoda: Cephalobidae). Nematology 1:591–612.

[ref009] Decraemer W. and Geraert E. 2006 Ectoparasitic nematodes In Perry R. N. and Moens M. (Eds). Plant Nematology CABI, Wallingford, 153–184.

[ref010] Decraemer W. and Hunt D. 2006 Structure and classification In Perry R. N. and Moens M. (Eds). Plant Nematology CABI, Wallingford, 3–32.

[ref011] Ebsary B. A. 1981 *Neobakernema* n. gen. (Nematoda: Criconematidae) with an emendation of *Bakernema* Wu, 1964. Canadian Journal of Zoology 59:2215, R. N., Perry and M., Moens (Eds). p. 2216.

[ref012] Geraert E. 2010 The Criconematidae of the World. Identification of the Family Criconematidae (Nematoda) Academia Press, Gent.

[ref013] Hall T. A. 1999 BioEdit: a user-friendly biological sequence alignment editor and analysis program for Windows 95/98/NT. Nucleic Acids Symposium Series 41:95–98.

[ref014] Ivanova T. S. 1984 New species of nematode *Nothocriconemoides crenulatus* sp.n. (Nematoda: Criconematidae) from mountain regions in Tadzhikistan. Izvestiya Akademii Nauk Tadzhikiskoi SSR Otdelenie Biologicheskikh Nauk 20–22.

[ref015] Jahanshahi-Afshar F., Pourjam E. and Pedram M. 2019 New morphological observations on *Neolobocriconema serratum* (Khan & Siddiqi, 1963) Mehta & Raski, 1971 (Rhabditida: Criconematidae). Nematology 21:419–434.

[ref016] Jenkins W. R. 1964 A rapid centrifugal-flotation technique for separating nematodes from soil. Plant Disease Reporter 48:692.

[ref017] Joyce S., Reid A., Driver F. and Curran J. 1994 Application of polymerase chain reaction (PCR) methods to identification of entomopathogenic nematodes In Burnell A. M., Ehlers R. U. and Masson J. P. (Eds). COST 812 Biotechnology: Genetics of Entomopathogenic Nematode-bacterium Complexes. Proceedings of Symposium & Workshop St. Patrick’s College, Maynooth, Co. Kildare, European Commission, DG XII, Luxembourg, 178–187.

[ref018] Khan E. and Siddiqi M. R. 1963 *Criconema serratum* n. sp. (Nematoda: Criconematidae), a parasite of peach trees in Almore, North India. Current Science 32:414–415.

[ref019] Katoh K. and Standley D. M. 2013 MAFFT multiple sequence alignment software version 7: improvements in performance and usability. Molecular Biology and Evolution 30:772–780. 23329690 10.1093/molbev/mst010 PMC3603318

[ref021] Loof P. A. A. 1988 “Identification of Criconematids”, in Fortuner R. (Ed.). Nematode Identification and Expert System Technology Plenum Press, New York, NY, 139–152.

[ref022] Luc M. 1959 Nouveaux Criconematidae de la zone intertropicale (Nematoda:Tylenchida). Nematologica 4:16–22.

[ref023] Maas P. W. T., Loof P. A. A. and De Grisse A. 1971 *Nothocriconemoides lineolatus* (n. gen., n. sp. Nematoda:Criconematidae). Mededelingen Fakuleti Land Bouwwetenschappen Gent 36:711–715.

[ref024] Maria M., Cai R., Castillo P. and Zheng J. 2018a Morphological and molecular characterization of *Hemicriconemoides paracamelliae* sp. n. (Nematoda: Criconematidae) and two known species of *Hemicriconemoides* from China. Nematology 20:403–422.

[ref025] Maria M., Powers T. O., Tian Z. and Zheng J. 2018b Distribution and description of criconematids from Hangzhou, Zhejiang Province, China. Journal of Nematology 50:183–206. 10.21307/jofnem-2018-010 PMC6909330 30451437

[ref026] Maria M., Cai R., Subbotin S. A. and Zheng J. 2019 Description of *Discocriconemella sinensis* n. sp. (Nematoda: Criconematidae) from the rhizosphere of *Camellia sinensis* in China. Nematology 21:779–792.

[ref027] Mehta U. K. and Raski D. J. 1971 Revision of the genus *Criconema* Hofmänner & Menzel, 1914 and other related genera (Criconematidae: Nematoda). Indian Journal of Nematology 1:145–198.

[ref028] Micoletzky H. 1922 Die freilebenden Erd-Nematoden: mitbesonderer Berücksichtigung der Steiermark und der Bukowina, zugleich mit einer Revision sämtlicher, nicht mariner, freilebender Nematoden in Form von Genus-Beschreibungen und Bestimmungsschlüsseln. Archiv für Naturgeschichte Berlin Abteilung A 87:1–650.

[ref029] Micoletzky H. 1925 Die freilebenden Susswasser and Moornematoden Danemarks. Det Kongelige DanskeVidenskabelige Seldskap Skrifter, Naturvidens-Kabelige og Mathematiske Afdeling 8:57–310.

[ref030] Olson M., Harris T., Higgins R., Mullin P., Powers K., Olson S. and Powers T. O. 2017 Species delimitation and description of *Mesocriconema nebraskense* n. sp (Nematoda: Criconematidae), a morphologically cryptic, parthenogenetic species from North American Grasslands. Journal of Nematology 49:42–66. 28512377 10.21307/jofnem-2017-045 PMC5411254

[ref031] Orton Williams K. J. 1981 Revision of the genus *Discocriconemella* De Grisse & Loof, 1965 and the erection of the new genus *Acrozostron* (Nematoda: Criconematoidea). Systematic Parasitology 2:133–138.

[ref032] Powers T. O., Harris T., Higgins R., Sutton L. and Powers K. S. 2010 Morphological and molecular characterization of *Discocriconemella inarata*, an endemic nematode from North American native tallgrass prairies. Journal of Nematology 42:35–45. 22736835 PMC3380506

[ref046] Powers T. O., Bernard E. C., Harris T., Higgins R., Olson M., Lodema M., Mullin P., Sutton L. and Powers K. S. 2014 COI haplotype groups in *Mesocriconema* (Nematoda: Criconematidae) and their morphospecies associations. Zootaxa 3827:101–146, DOI: 10.11646/zootaxa.3827.2.1. 10.11646/zootaxa.3827.2.1 25081151

[ref033] Powers T., Harris T., Higgins R., Mullin P. and Powers K. 2017 An 18S rDNA perspective on the classification of Criconematoidea. Journal of Nematology 49:236–244. 29062146 PMC5644916

[ref034] Raski D. J. 1952 On the morphology of *Criconemoides* Taylor, 1936, with descriptions of six new species (Nematoda: Criconematidae). Proceedings of the Helminthological Society of Washington 19:85–99.

[ref035] Ronquist F., Teslenko M., van der Mark P., Ayres D. L., Darling A., Höhna S., Larget B., Liu L., Suchard M.A. and Huelsenbeck J. P. 2012 MRBAYES 3.2: Efficient Bayesian phylogenetic inference and model selection across a large model space. Systematic Biology 61:539–542. 22357727 10.1093/sysbio/sys029 PMC3329765

[ref036] Seinhorst J. W. 1959 A rapid method for the transfer of nematodes from ﬁxative to anhydrous glycerin. Nematologica 4:67–69.

[ref037] Siddiqi M. R. 2000 Tylenchida: Parasites of Plants and Insects 2nd ed., CABI Publishing, Wallingford, 833.

[ref039] Subbotin S. A., Madani M., Krall E., Sturhan D. and Moens M. 2005 Molecular diagnostics, taxonomy, and phylogeny of the stem nematode *Ditylenchus dipsaci* species complex based on the sequences of the internal transcribed spacer-rDNA. Phytopathology 95:1308–1315. 18943362 10.1094/PHYTO-95-1308

[ref040] Taylor A. L. 1936 The genera and species of the Criconematinae, a sub-family of the Anguillulinidae (Nematoda). Transactions of the American Microscopical Society 55:391–421.

[ref041] Vovlas N. 1992 Taxonomy of *Discocriconemella* (Nematoda: Criconematoidea) with a redescription of *D. mauritiensis* . Journal of Nematology 24:391–398. 19283014 PMC2619297

[ref042] Wouts W. M. 2006 Criconematina (Nematoda: Tylenchida) Fauna of New Zealand 55. Lincoln, New Zealand, Landcare Research, p. 232.

[ref043] Xiang M. C., Xiang P. A., Jiang X. Z., Duan W. J. and Liu X. Z. 2010 Detection and quantification of the nematophagous fungus *Hirsutella minnesotensis* in soil with real-time PCR. Applied Soil Ecology 44:170–175.

[ref044] Ye W., Giblin-Davis R. M., Braasch H., Morris K. and Thomas W. K. 2007 Phylogenetic relationships among *Bursaphelenchus* species (Nematoda: Parasitaphelenchidae) inferred from nuclear ribosomal and mitochondrial DNA sequence data. Molecular Phylogenetics and Evolution 43:1185–1197. 17433722 10.1016/j.ympev.2007.02.006

[ref045] Zheng J., Subbotin S. A., He S., Gu J. and Moens M. 2003 Molecular characterization of some Asian isolates of *Bursaphelenchus xylophilus* and *B. mucronatus* using PCR-RFLPs and sequences of ribosomal DNA. Russian Journal of Nematology 11:17–22.

